# Aggregate morphing of self-aligning soft active disks in semi-confined geometry

**DOI:** 10.1038/s41598-024-77219-7

**Published:** 2024-11-11

**Authors:** Anshika Chugh, Soumen De Karmakar, Rajaraman Ganesh

**Affiliations:** 1 Institute for Plasma Research, Bhat, Gandhinagar, 382428 India; 2 Homi Bhabha National Institute, Training School Complex, Anushaktinagar, Mumbai, 400094 India; 3 Institute for Theoretical Physics IV, University of Stuttgart, Heisenbergstr. 3, 70569 Stuttgart, Germany

**Keywords:** Self-assembly, Physics, Biological physics

## Abstract

We study the dependence of alignment and confinement on the aggregate morphology of self-aligning soft disks(particles) in a planer box (two dimensional) geometry confined along *y* direction using Langevin dynamics simulations. We show that when the box width decreases, the aggregate wall accumulation becomes non-uniform and displays non-monotonic behaviour in terms of phase behavior and height of these aggregates with an increase in alignment strength. Additionally, we identify two distinct categories of wall aggregates: layered and non-layered structures each exhibiting distinct local structural properties. For non-layered structures, local speed of the particles stay nearly constant as we move away from the boundary, while for layered structures, it increases with distance from the boundary. Our analysis shows that active pressure difference is a useful indicator for different aggregate morphologies and the peaks in the pressure curve are indicative of the average and minimum height of the structure.

## Introduction

Motility induced phase separation (MIPS)^1^ is an interesting collective phenomenon that is unique to active matter systems. Due to particles’ self-propulsion, uniformly distributed particles tends to spontaneously phase separate into dense and dilute regions. However, this phase separation requires sufficiently high motility and relatively high packing fraction. Interestingly, under certain alignment^2–4^ rules between particles, MIPS can been observed even at low packing fraction. Studies on MIPS has a rich literature encompassing investigations into the dynamics within both non-inertial^5,6^ and inertial limits^7–9^, with and without external alignment among particles^10,11^, and there have been few recent studies with soft particles^9,12,13^. Majority of these studies focus on the bulk behavior and properties in unbounded or doubly-periodic systems. The structural and dynamical properties of clusters or aggregates generated in MIPS have been intensively studied in bulk systems^5,14^. These studies have yielded important insights into the emergent properties^2,3,5,11^ of active particles. However, studying collective phenomenon in confined and crowded environments is crucial, as various active entities are naturally found in confined spaces such as soil (porous media)^15^, narrow channels (blood vessels)^16,17^ and biofilms^18^. Understanding how active particles behave in confined spaces has potential applications in sorting and drug delivery^19,20^.

Active particles do not immediately bounce off the boundaries due to their persistence. Consequently, the particles are accumulated at the boundaries that has been observed in experiments^21–23^ and in numerical simulations^24–26^. For example, it gives insights on the pattern formation of bacteria in natural settings^18^. Moreover, boundary shape strongly influences the spatial distribution of the particles when the size of the confined space is smaller than the persistence length^25^. Other studies demonstrate the effect of particle-boundary interactions^27^, and particle-particle interactions^28^ on the accumulation of particles at the boundary which leads to rectification effects and spontaneous segregation of the particles. A recent study on MIPS in confined environments include that in porous environment such as porous walls, where the clusters undergo a morphological transition from a uniform accumulation at walls to accumulation at specific locations on the wall^29,30^. However, a less attention has been given to considering particle alignment as an alternative approach to influence the dynamics of wall accumulation.

The present study investigates how particle alignment affects the dynamics of active particles in a semi-confined planer geometry. We consider a non-reciprocal alignment interaction between the self-propelled particles that reorient the self-propulsion direction of the particles along their inter-particle separation and towards each other^4,31,32^. We consider a low packing fraction regime where the alignment interaction is known to exhibit MIPS in bulk^4,32^, while MIPS is not observed for uncoordinated or non-aligning self-propelled particles^33^. We majorly focus on soft particles^9,13^, which is more relevant in the context of biological active species that exhibit substantial deformation in their shapes on collision compared to significantly low deformation of their synthetic counterpart. Additionally, we consider a small inertia of the particles as larger inertia is known to disfavor MIPS. A detailed investigation of the role of inertia, particle’s softness and alignment interaction on MIPS in bulk is presented in Ref.^31^. We show that the soft particles with the considered alignment interaction exhibit a rich morphology of wall aggregation in the semi-confinement. We characterize the aggregate morphology and present it in a phase diagram in the space of alignment strength and confinement width. We show that the wall aggregation becomes non-uniform with decrease in the confinement width, while it shows a non-monotonic dependence on the alignment interaction strength. Additionally, our results show that the height of the aggregates exhibits a non-monotonic dependence on the alignment strength. We find that the active pressure difference between the normal and the tangential components is an important indicator for the average and the minimum height of the wall aggregates.

In “Model” section we introduce our model and provide the model parameters, in “Results and discussions” section we provide the results and discuss them, finally we conclude in “Conclusions” section.

**Fig. 1:**
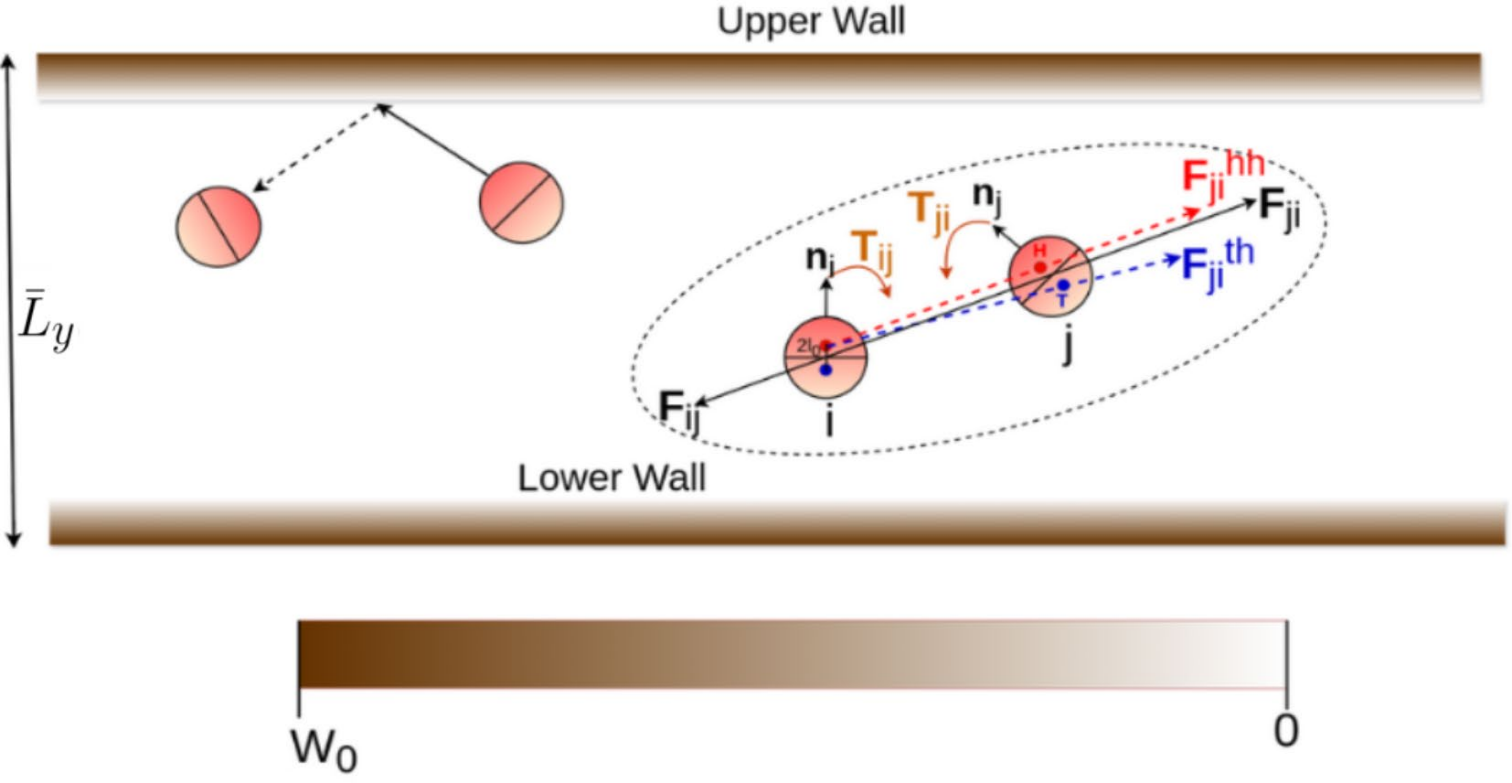
Illustrative diagram of particle-wall interaction and particle–particle interaction. Color bar denotes the strength of potential at the wall. In the hard wall limit, only the particles very close to the wall feel high repulsive force and are reflected from the wall boundary. Solid and dashed arrow show the particle trajectory before and after collision respectively. Particle–particle interaction with the alignment model^4^ is shown in dashed circle. The head and tail of the one disk separately interacts with the two hemispheres of the other disks. The head and tail force centers, denoted by the red and blue dots, respectively, are located $2 l_0$ distance apart. The head force center of the *i*th disk interacts with the head (indicated by a red arrow) and the tail (indicated by a blue arrow) force centers of the *j*th disc, giving rise to a resultant force represented by a black arrow. This results in nonreciprocal torque ($T_{ij}$) which aligns the self-propulsion axis ($n_i$) of both of the disks along the inter-particle separation ${\textbf {r}}_{ij}$ and towards each other. $\bar{L}_y$ denotes the dimensionless confinement width along the *y* direction.

## Model

We study a system of self-aligning soft active disks of effective diameter $\sigma$ in a semi-confined box, periodic along *x* and bounded along *y*, of aspect ratio $L_x / L_y$. We consider a non-reciprocal alignment interaction between the particles that reorient the self propulsion directions of the particles along their inter-particle separation and towards each other^4,32^. The reorientation mechanism is depicted in Fig. 1. To obtain the resultant alignment interaction, the particles of mass *m* are subdivided into two hemispheres- head and tail- with respective force centers separated at a small distance $2l_0$, as shown in Fig. 1. The head and tail force centers of the particles separately interacts with the two force centers of the other particles with a modified form of the Yukawa potential $U^{ht}_{ij} = U^{ht}_0 \displaystyle {e^{-(r^{ht}_{ij}-\sigma )/ \lambda }}/r^{ht}_{ij}$. $\lambda$ controls the softness of the disks^9^ and the criteria $\tau _0 = l_0(U^{tt}_0 - U^{hh}_0) > 0$ give rise to the torque which non-reciprocally aligns the particles along their inter-particle separation and towards each other^4,31^, as shown in Fig. 1. It has been shown previously^4,32^ that this alignment model gives rise to MIPS at area fractions well below the critical threshold. Hence, we consider a low area fraction $\phi = N \pi \sigma ^2 / (4 Lx L_y) = 0.15$, where MIPS of uncoordinated self-propelled particles is not observed^33^.

Figure 1 shows an illustrative diagram of particle-wall interaction where the particles interact with the wall using the potential form^34^$W(y) = W_0 (1 + tanh \hspace{0.1cm} q(y - 0.5 L_y))\forall y \ge 0$ and $W(y) = W_0 (1 - tanh \hspace{0.1cm} q(y + 0.5 L_y))\forall y < 0$, and color bar indicates the strength of potential. The strength $W_0$ is chosen such that it is much greater than the individual particle’s total energy to ensure confinement of the particles along the *y* direction. The parameter *q* controls the steepness of the boundary. *q* is fixed to a large value, $q = 10$, to mimic the hard wall limit, where only the closest layer to the boundary experiences large repulsive force. Using the in-house developed Molecular Dynamics solver MPMD^4,9,35^, we solve the following set of underdamped Langevin equations.

1 $$\begin{aligned} m \ddot{{\textbf {r}}_i}&= -\gamma \dot{{\textbf {r}}_i} + \sqrt{2 \gamma ^2 D} \pmb {\xi }_i + \sum _j {\textbf {F}}_{ij} + \gamma \text {v}_0 {\textbf {n}}_i - \pmb {\nabla }W(y_i), \end{aligned}$$

2 $$\begin{aligned} \dot{{\textbf {n}}}_i&= \sqrt{2 D_r} \pmb {\zeta }_i \times {\textbf {n}}_i + \frac{1}{\gamma _r} \sum _j {\textbf {T}}_{ij} \times {\textbf {n}}_i, \end{aligned}$$

where ${\textbf {F}}_{ij} = U_0 \frac{e^{-(r_{ij}-\sigma )/\lambda }}{r_{ij}^2} (\frac{1}{r_{ij}} + \frac{1}{\lambda }) {\textbf {r}}_{ij}$ is the resultant force experienced by the center of mass of the particle *i* due to its head and tail component’s soft interaction with the head and the tail component of the particle *j* in limiting approximation $r_{ij} \gg 2l_0$. ${\textbf {T}}_{ij} = \frac{\tau _0}{U_0} {\textbf {F}}_{ij} \times {\textbf {n}}_j$ is the torque that naturally appears out of the resultant soft interactions on the two force centers of the particles. $U_0$ is the resultant interaction strength given by $U_0 = U^{hh}_0 + 2 U^{ht}_0 + U^{tt}_0$. The derivation of the resultant force, ${\textbf {F}}_{ij}$ and the torque ${\textbf {T}}_{ij}$ can be found in Ref.^31^. ${\textbf {n}}_i$ denotes the propulsion direction pointing from the head and perpendicular to the line dividing the disk into two hemispheres. Here, $\gamma$ and $\gamma _r$ are dissipation coefficients, *D* and $D_r$ are diffusion coefficients. The suffix r denotes rotational parameter. $\pmb {\xi }_i$ and $\pmb {\zeta }_i$ are Gaussian white noise.

We measure length, time and energy in units of $\sigma$, $1/D_r$ and $k_BT$ respectively. Therfore, the reduced parameters are the inertial parameter $M = \frac{m / \gamma }{1 / D_r}$, the resultant interaction strength $\Gamma = \frac{U_0 / \sigma }{k_BT}$, the softness parameter $\kappa = \sigma / \lambda$, the Peclet number $P_e = \frac{1/D_r}{\sigma / \text {v}_0}$, and the reorientational interaction strength $\Lambda = \frac{1 / D_r}{\gamma _r \sigma ^2 / \tau _0 }$. The corresponding reduced equations are:

3 $$\begin{aligned} M \ddot{{\textbf {r}}_i}&= - \dot{{\textbf {r}}_i} + \sqrt{2} \pmb {\xi }_i + \Gamma \sum _j \frac{e^{-\kappa (r_{ij} -1)}}{r_{ij}^2}\left( \frac{1}{r_{ij}} + \kappa \right) {\textbf {r}}_{ij} + P_e {\textbf {n}}_i - \pmb {\nabla }W(y_i), \end{aligned}$$

4 $$\begin{aligned} \dot{{\textbf {n}}}_i&= \left[ \sqrt{2} \pmb {\zeta }_i + \Lambda \sum _j \frac{e^{-\kappa (r_{ij} -1)}}{r_{ij}^2}\left( \frac{1}{r_{ij}} + \kappa \right) {\textbf {r}}_{ij} \right] \times {\textbf {n}}_i . \end{aligned}$$

A full scale parameter study for *M*, $\Gamma$, $\kappa$ and $P_e$ is reported in Ref. ^31^. The study reveals that increasing inertia, *M* and softness (decreasing $\kappa$) suppresses MIPS, while $P_e$ promotes MIPS, enabling its occurrence even at relatively low values of $\kappa$. Additionally, as $\Gamma$ increases, MIPS exhibits re-entrant behavior for a given $\kappa$, *M* and $P_e$. In the present study, we consider soft disks with finite inertia and repulsive interactions of intermediate strength. The considered fixed parameters are: $M = 0.05$, $\Gamma = 25.0$, $P_e = 75.0$. We mainly use $\kappa = 5$ (soft particle limit), but we also show results for sufficiently large $\kappa = 18$ (hard particle limit) for comparison. The value of the strength of wall potential, $W_0$ is fixed to 1000 which is sufficient to confine the particles within the walls for the given parameters. We define ${\bar{L}_y} = L_y / l_p$ as the dimensionless confinement width along the *y* direction. $l_p$ is the persistence length of the active particle which in terms of Peclet number can be written as $l_p = \sigma P_e$. Thus, ${\bar{L}_y} = L_y / (\sigma P_e)$. In the absence of the alignment strength, system remains homogeneous at the chosen value of the packing fraction. The system variables are alignment strength $\Lambda$ and dimensionless confinement width ${\bar{L}_y}.$ The parameter, $\Lambda$ signifies the strength of orientation of the self-propulsion disks towards each other and ${\bar{L}_y}$ signifies the narrowness of the semi-confined geometry. We study the dynamics of $N = 48,400$ particles in two dimensions in the parameter space ${\bar{L}_y} - \Lambda$ with confinement along *y* direction. As the number of particles *N* and the area fraction $\phi$ are fixed, variation in dimensionless confinement width ${\bar{L}_y}$ implies change in box length along *x* direction, i.e, the change in the aspect ratio of the box. However, we observe similar results by varying the number of particles and keeping the aspect ratio same, at the same area fraction. In other words, the aspect ratio has negligible effect on the system as long as the separation distance between the walls (confinement) remains the same. All the simulations are performed for 5000 units of time with step size $\delta t = 0.0005$. This amounts to $10^7$ simulation steps. The simulation runtime of $t = 5000$ time units are sufficient to obtain the steady state, where the number of particles in the different motility induced phase separated states is nearly constant with time and consequently steady value of the quantities of interest (such as aggregation length and height of the clusters) is reached. To obtain the averages, the quantities of interest are summed over 200 steady state configurations.

## Results and discussions

**Fig. 2:**
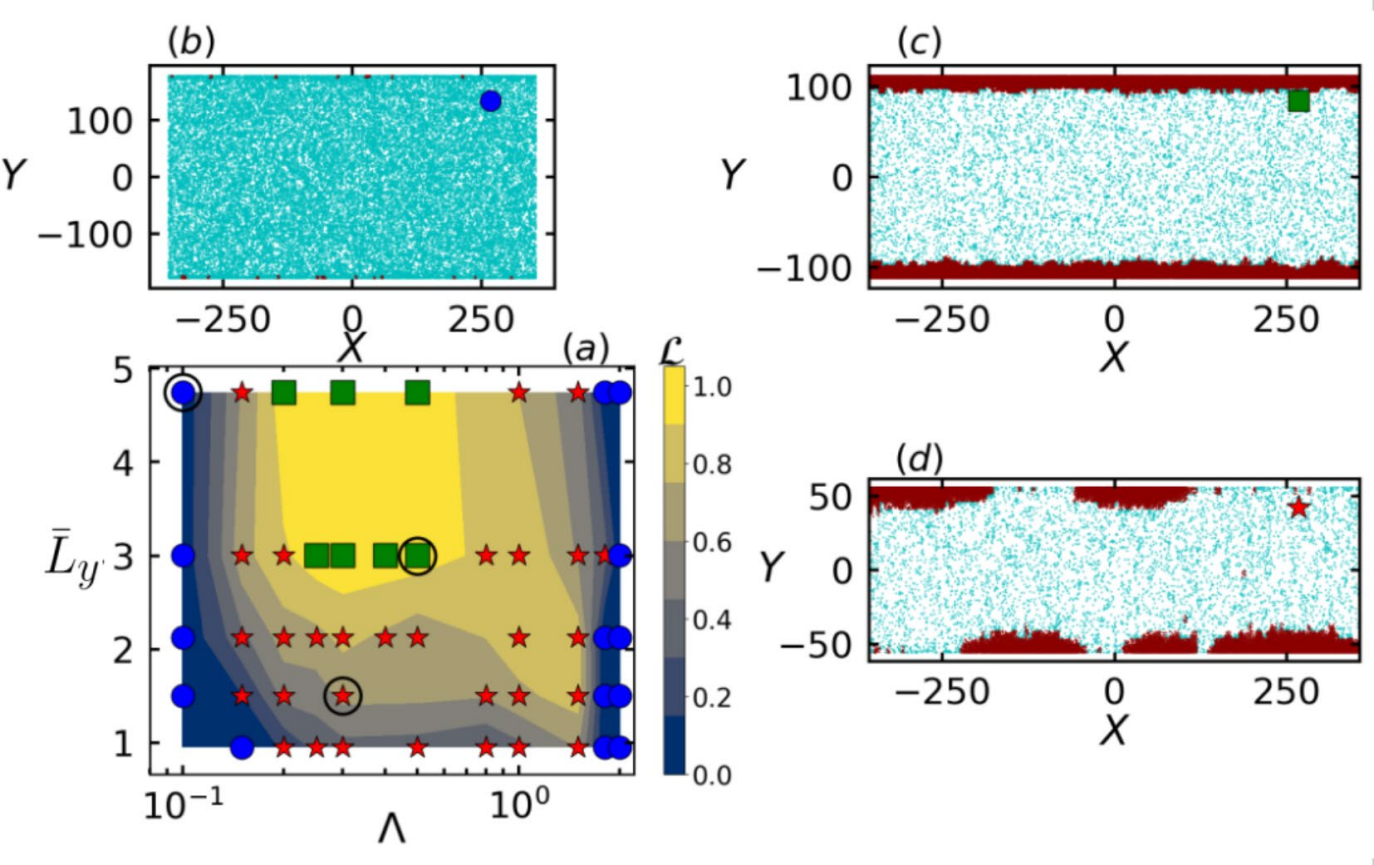
(**a**) Phase Diagram in the parameter space of dimensionless confinement width ${\bar{L}_y}$ and alignment strength $\Lambda$. Three different states can be distinguished as (**b**) homogeneous state (blue circle) with $\mathcal {L} \le 0.1$, (**c**) uniform aggregation state (green rectangle) with $\mathcal {L} \ge 0.9$ and (**d**) non-uniform aggregation state (red star) with $0.1< \mathcal {L} < 0.9$. Red and blue particles constitute part of dense and dilute phases, respectively.

Figure 2a summarizes the effect of the dimensionless confinement width ${\bar{L}_y}$ and alignment strength $\Lambda$ in the form of a phase diagram in the space of ${\bar{L}_y}$ and $\Lambda$. The phase diagram is divided into distinct regions according to the value given by aggregation length $\mathcal {L}$ that serves as an identifier for different aggregates morphology (see “Appendix” section for the definition). For negligible accumulation of particles along the walls, where $\mathcal {L} \le 0.1$, the state is called as homogeneous state (Fig. 2b). When the particles form clusters along the whole length of the wall, the state is called uniform aggregation state, where $\mathcal {L} \ge 0.9$ (Fig. 2c). When the accumulation or the formation of clusters is at specific sites on the wall, leaving a portion of the wall unoccupied, then the state is called non-uniform aggregation state, where $\mathcal {L}$ lies between 0.1 and 0.9 (Fig. 2d). The distinction between the three phases is qualitative where an approximation of 10 percent variation is chosen as the criteria for identifying the three phases^30^.

We observe that the system displays reentrant behavior; the system is found in the homogeneous state at low and high alignment strengths, which is similar to what is observed in doubly periodic systems without the walls, i.e. in the bulk^4^. However for confined systems, cluster formation occurs at wall boundaries as the walls act as the potential nucleation sites for the formation of the clusters^36^. Particles approaching the walls get stuck until they turn their self-propulsion direction away from the wall, i.e., self-trapped^4^. This effectively slows down the particles and ensues wall accumulation. For low $\Lambda$, nucleation occurs only at the wall boundaries. As the alignment strength increases, large number of small clusters appear in between the walls which eventually dissolve and get accumulated at the wall boundaries. Figure 2a shows that at low and high values of the alignment strength, the occurrence of homogeneous state is independent of the confinement width ${\bar{L}_y}$. That is, despite the presence of the walls, which act like nucleation sites, only a small fraction of the self-propelled particles accumulate at the walls and the densities of the particles are not large enough to ensue cluster growth or the wall aggregation in the limit of small and large alignment strengths. At small $\Lambda$, the strength of the alignment torque is extremely small to trigger any cluster growth and at large strengths of alignment torque, a large number of small clusters appear which collide and break away due to increased reorientation frequency of the particles, and the system appears to be homogeneous. It demonstrates that the formation of a clusters or aggregates is linked to the alignment between the disks while location or the spread of the clusters is dominated by the interplay of alignment strength and the confinement width.

Uniform aggregation state is observed at intermediate values of $\Lambda$ and for ${\bar{L}_y} > 2.0$. With decrease in confinement width, the pipe geometry becomes narrower which increases the effective collisions between the disks along the path perpendicular to the walls. Hence, due to this space inhomogeneity of the collisions, where the probability of collisions perpendicular to the walls is greater than the collisions in the direction parallel to the walls, the morphology of the aggregates tends to change to non-uniform aggregation state. This effect can be seen in Videos 1 and 2, where the shape of the transient clusters shifts from circular to elongated with decrease in confinement width and increase in alignment strength as the particles joins from above and below rather than particles joining the cluster uniformly from all sides. For the given $P_e$, with decrease in confinement width, particles reach the wall faster which increases the probability of collisions with the particles on the way towards the wall. Hence, the nucleation sites grow quickly which trigger the formation of large number of small clusters in between the walls. Due to frequent collisions with decreased confinement width and increased alignment strength, particles do not get enough time to quickly move out of transient clusters at the wall and therefore exhibits in-homogeneous particle accumulation at the walls, whereas the particles get more time due to comparatively lower collision frequencies at larger ${\bar{L}_y}$ to move out of the transient clusters at the walls and asymptotically form uniform accumulation.

**Fig. 3:**
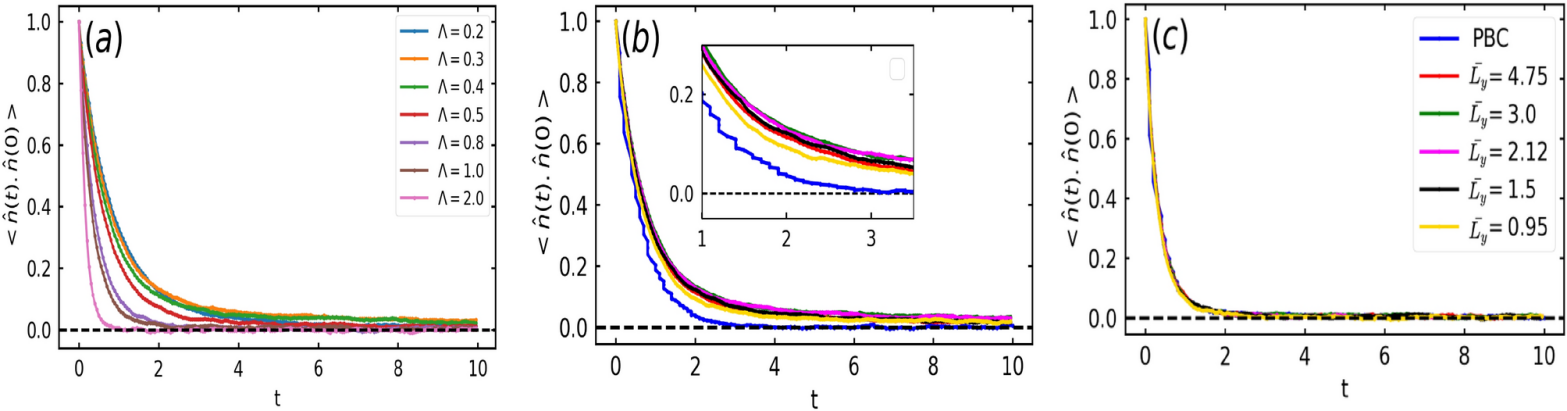
(**a**) Auto-correlation of the self-propulsion direction $<\textbf{n}_i(t) \cdot \textbf{n}_i (t = 0)>$ for various values of $\Lambda$ at fixed ${\bar{L}_y} = 3.0$. (**b, c**) Comparison of the auto-correlation of the self-propulsion direction in semi-confined geometry at various ${\bar{L}_y}$ with doubly periodic boundary system (blue line) at fixed (**b**) $\Lambda = 0.3$ and (**c**) $\Lambda = 1.0$.

Figure 3 captures the effect of increased collisions upon increase in $\Lambda$ and decrease in ${\bar{L}_y}$ using the autocorrelation of self-propulsion direction, which decays faster upon increase in $\Lambda$ (Fig. 3a) and decrease in ${\bar{L}_y}$ (Fig. 3b). However, at a sufficiently large $\Lambda$, the strength of alignment torque becomes so large that the decay of the autocorrelation becomes independent of the wall separation (Fig. 3c). Furthermore, we also compare the autocorrelation in this confined geometry at various confinement widths to the autocorrelation in doubly periodic system (PBC) for small and large values of $\Lambda$ (Fig. 3b,c). It is observed that the autocorrelation decays slower in comparison to PBC for small $\Lambda$ and the decay rate approaches to that of PBC at large $\Lambda$ as shown in Fig. 3b,c respectively. This can be explained on the basis of layering of particles, as discussed in the following paragraphs.

**Fig. 4:**
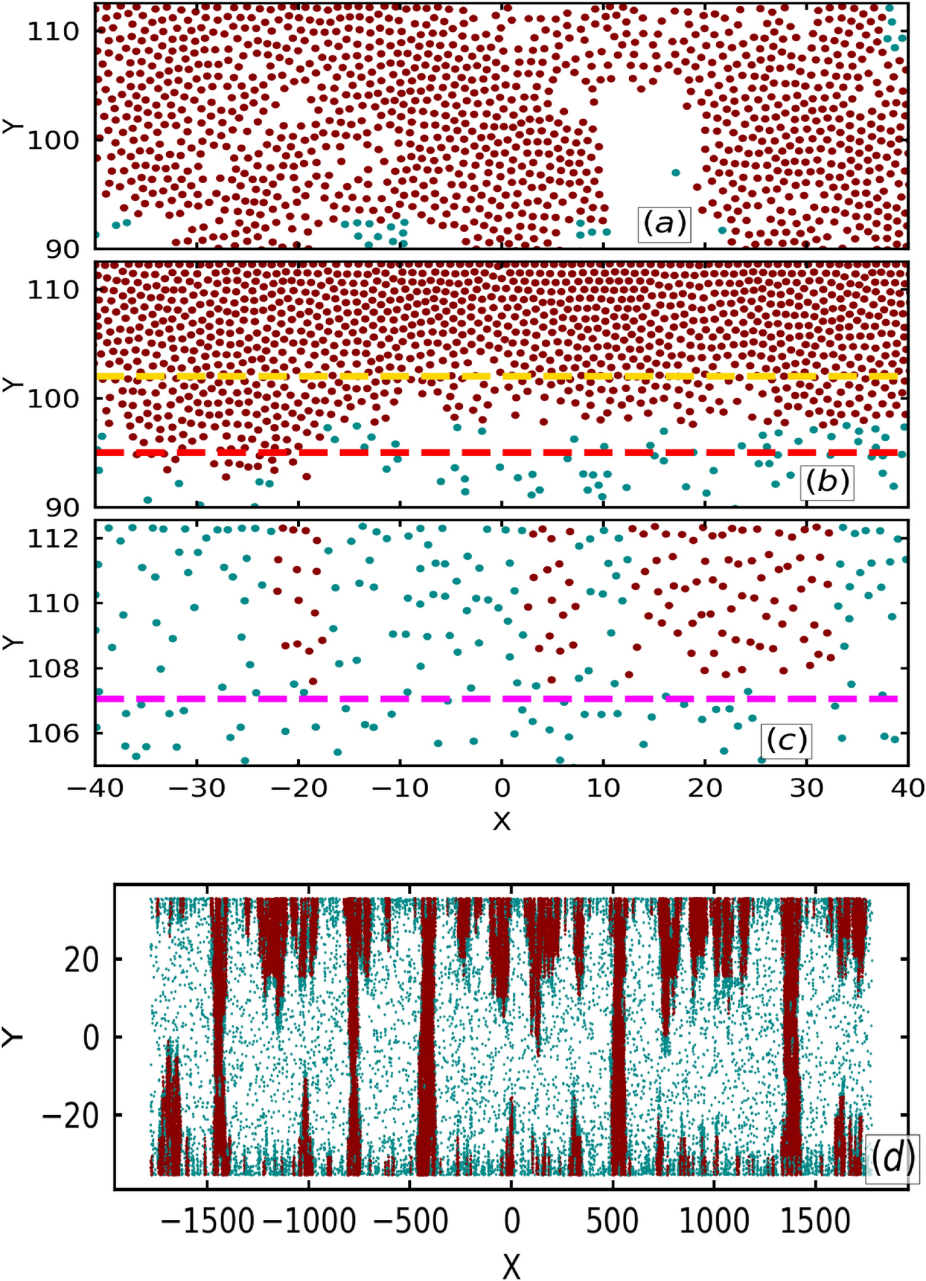
Configuration of particles for (**a**) $\Lambda = 1.0$, (**b**) $\Lambda = 0.3$ and (**c**) $\Lambda = 0.1$ at fixed ${\bar{L}_y} = 3.0$. (d) Configuration for ${\bar{L}_y} = 0.95$ and $\Lambda = 1.0$. Particles in dense and dilute phase are shown in red and blue, respectively. Layered and non-layered structures are observed for intermediate and high $\Lambda$ values, respectively. Dashed lines corresponds to a particular distance from the wall as shown in Fig. 5c. Magenta and red dashed lines are positions of average height of the structure and yellow dashed line is the minimum height of the structure. Only a fraction of geometry is shown for configurations in (**a–c**).

Figure 4 shows distinct structural patterns at different values of $\Lambda$, namely $\Lambda = 1.0$ (Fig. 4a), $\Lambda = 0.3$ (Fig. 4b), $\Lambda = 0.1$ (Fig. 4c) for the fixed ${\bar{L}_y} = 3.0$. At small value of ${\bar{L}_y} = 0.95$, the non-uniform aggregates are found to bridge^37^ the walls, which is shown in Fig. 4d for $\Lambda = 1.0$. Depending on the local arrangement of particles, these structures are distinguished as layered and non-layered structures^28^. At intermediate values of $\Lambda$, we observe layered structures (Fig. 4b) while at larger $\Lambda$, the layering if found to vanish (Fig. 4a). Due to increased alignment strength between the particles, a large number of small dynamic clusters appear in between the walls and these clusters tend to join the clusters at the wall via a bridge of clusters closest to them which leads to random local arrangement of the particles also leaving gaps or dilute regions in between dense regions as shown in Fig. 4a. Thus, layering of particles close to the wall does not happen. For ${\bar{L}_y} < 1$, these elongated clusters from the upper and the lower wall tend to join and form lane like structures (bridging) as shown in Fig. 4d. At intermediate value of the alignment strength, wall accumulation of the particles dominate over cluster formation in between the walls. Particles approaching the wall get trapped until they change their self-propulsion direction away from the wall, while the particles leaving the wall can move freely. This asymmetry leads to wall accumulation and slower decay of the self-propulsion direction autocorrelation in comparison to doubly periodic systems as shown in Fig. 3b. Thus, particles at the intermediate alignment strengths tend to form layers.

**Fig. 5:**
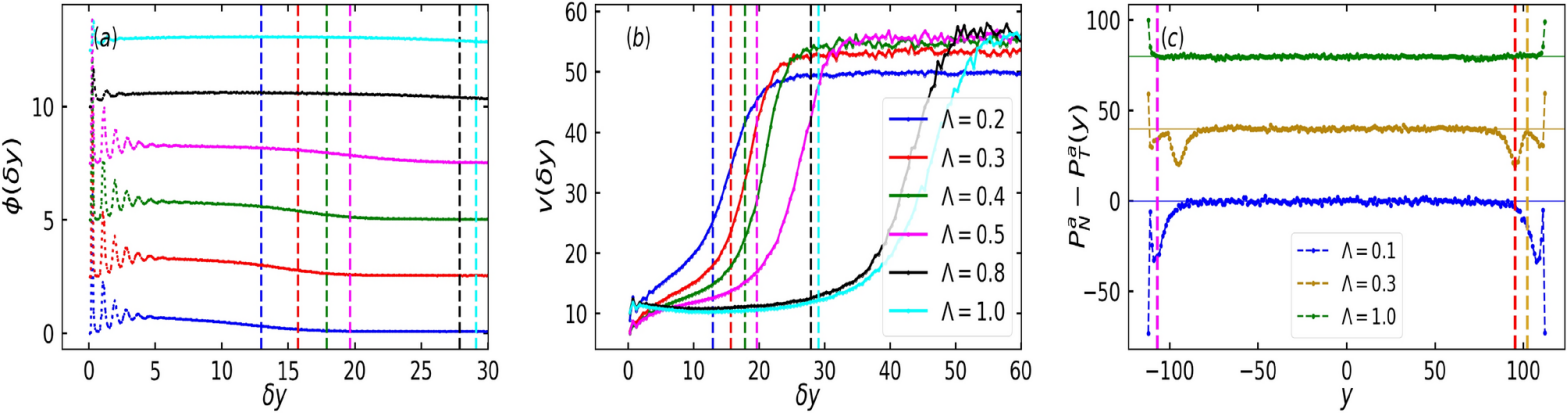
(**a**) Local area fraction $\phi (\delta y)$ and (**b**) local speed $\text {v} (\delta y)$ as a function of particles’ distance from the wall boundary $\delta y$ for fixed confinement width ${\bar{L}_y} = 3.0$. (**c**) Active pressure difference between the normal and the tangential components, $P^a_N - P^a_T$, as a function of distance from the wall $\delta y$ for different values of $\Lambda$ and fixed ${\bar{L}_y} = 3.0$. Local area fraction curves and the pressure curves are shifted along *y* by 2.5 and 40 units, respectively, for presentation purposes. The colored dashed lines in (**a, b**) show the position of the average height of the clusters for respective $\Lambda$. Yellow and red dashed lines in (**c**) are positions of minimum and average height of the structure for configuration given in Fig. 4b and magenta dashed line corresponds to the position of the average height of the structure for the configuration in Fig. 4c.

To visualize the the formation of layers close to the wall, we show the variation of local packing $\phi (\delta y)$ with distance from the wall $\delta y$ as shown in Fig. 5a. The peaks in the curves are reflective of the layered structure, which is present at low $\Lambda$ and vanishes at large $\Lambda$, when the alignment strength or torque takes over the wall slow down of the particles. The colored dashed line shows the position of the average height (see “Appendix” for calculation) of the clusters for respective $\Lambda$. In layered structures, particles are trapped and not free to move, while for non-layered structures the particles are highly mobile which leads to the apparent thermalisation of particles. Thus, as shown in Fig. 5b, for layered structure, i.e. for the corresponding value of $\Lambda$, the local speed, which is the average speed of the particles in the rectangular bin of width 1 and parallel to the wall gradually increases towards the dilute-dense interface or the average height of the structures, while for non-layered structure, where particles can move freely, the speed variation with the wall separation is nearly constant upto a distance of average height of structure and increases in the dilute region.

Figure 5c shows pressure difference for different values of $\Lambda$ for a given ${\bar{L}_y} = 3.0$. The contribution of the pressure is due to the motility of active particles and is given by $P^a_{\alpha \beta } (y) = \frac{1}{2 L_x \delta y} < \sum _{i \in \delta y} j_{i\alpha } \text {v}_{i\beta } >$ where $<...>$ indicates time averaging within rectangular bins parallel to the wall and of width $\delta y = 1$. ${\textbf {j}}_i = \gamma \text {v}_0 {\textbf {n}}_i / D_r$ stands for active impulse produced by active force $\gamma \text {v}_0 {\textbf {n}}_i$ and $\text {v}_i$ is the velocity of the *i*th particle. The non-zero diagonal components, $P^a_{xx}$ and $P^a_{yy}$ are respectively the tangential ($P^a_T$) and normal ($P^a_N$) components of the pressure tensor, $P^a_{\alpha \beta }$. However, calculating the difference between the normal and tangential components of pressure is of practical interest as one can obtain surface tension by evaluating the integrated difference of these components^38^. Active pressure difference, $P^a_N - P^a_T$ curve gives us the information about the layered structure, where the dips in the pressure curve are indicative of layered arrangement of particles (yellow curve in Fig. 5c). The longer dip (red dashed line) in the pressure difference curve gives the average height of the structures or the average distance of dense dilute interface and smaller dip (yellow dashed line) provides the minimum height of the structures. The absence of peaks in the pressure difference curve (green curve in Fig. 5c) suggests the absence of layers in the structures. Similarly, for the pressure difference curve of $\Lambda = 0.1$ (blue curve) in Fig. 5c, the position of the dip in the curve (magenta dashed line) represents the average height of the aggregates.

**Fig. 6:**
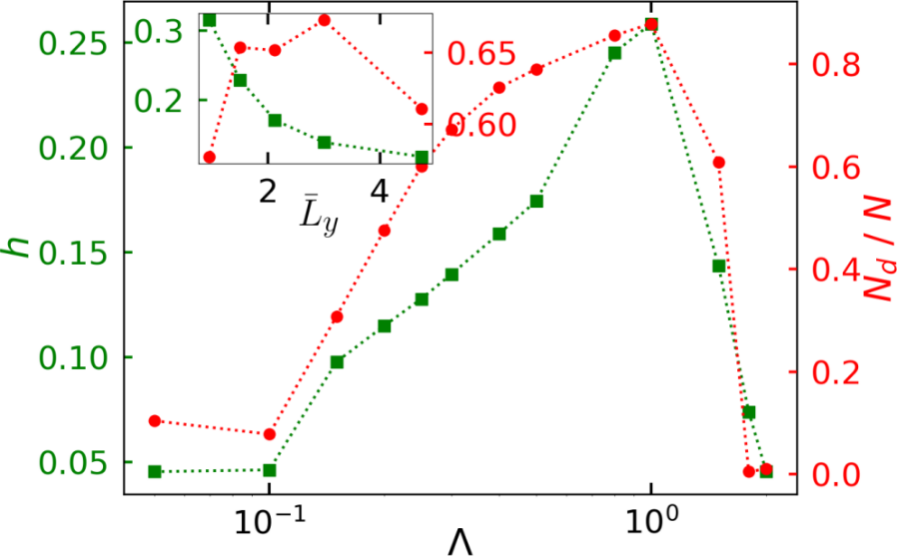
Wall aggregate height *h* (green square) and the fraction of the particles in the clusters $N_d / N$ (red circle) as a function of alignment strength $\Lambda$ for fixed dimensionless confinement width ${\bar{L}_y} = 3.0$. Inset shows the variation of these quantities with dimensionless confinement width ${\bar{L}_y}$ for a given alignment strength $\Lambda = 0.3$.

Figure 6 shows the steady state properties such as average height of the wall aggregation *h* (see Appendix for details) and the fraction of particles in the dense cluster $N_d / N$. As with increase in $\Lambda$, the number of transient clusters increase which eventually merge into large cluster at the wall boundary, therefore the fraction of particles in dense phase $N_d/ N$ increases with $\Lambda$. Since the height of wall aggregates is directly linked to the the cluster formation, therefore we observe a simultaneous increase in *h* and $N_d / N$. With further increase in $\Lambda$, the reorientation frequency becomes faster. Hence, the transient clusters form and break rapidly and only a fraction of them are able to reach the wall leading to decrease in height of the wall aggregates. Inset of Fig. 6 shows the effect of confinement width on the steady state properties for a given alignment strength $\Lambda = 0.3$. Due to elongated clusters with decrease in confinement width, the average height of the wall aggregate increases monotonically with decrease in confinement width. On the other hand, the variation of cluster fraction $N_d / N$ is found to be non-monotonic with decrease in confinement width.

**Fig. 7:**
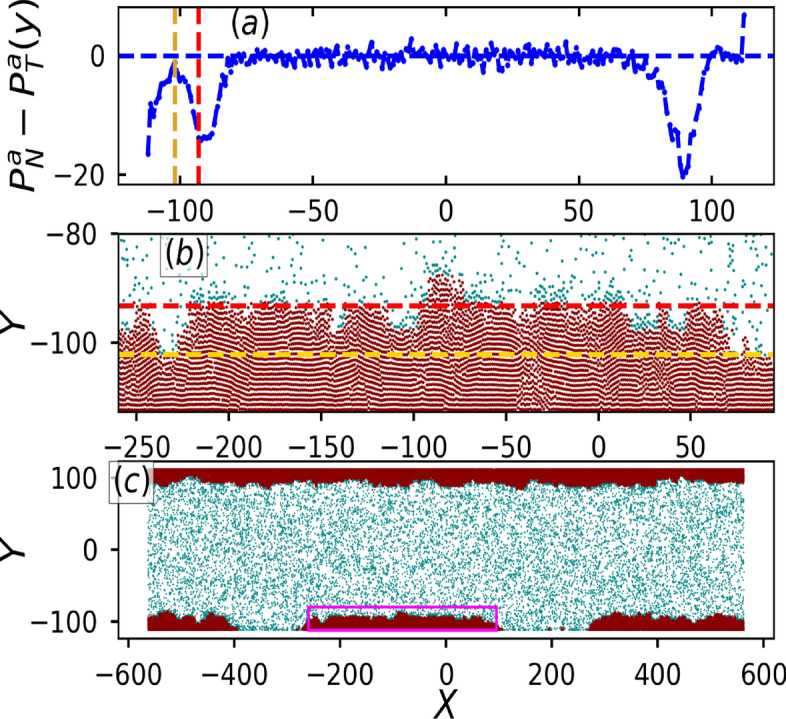
(**a**) Active pressure difference $P^a_N - P^a_T$ as a function of distance from the wall $\delta y$ for $\Lambda = 0.2$, ${\bar{L}_y} = 3.0$ and $\kappa = 18.0$ (hard particle limit). (**b**) Corresponding particle configuration at the lower wall of the box geometry defined by magenta color box in (**c**). Yellow and red dashed lines show the position of minimum and average height of the cluster respectively. (**c**) Full configuration of the system. Particles belonging to the dense and dilute state are colored red and blue, respectively.

**Fig. 8:**
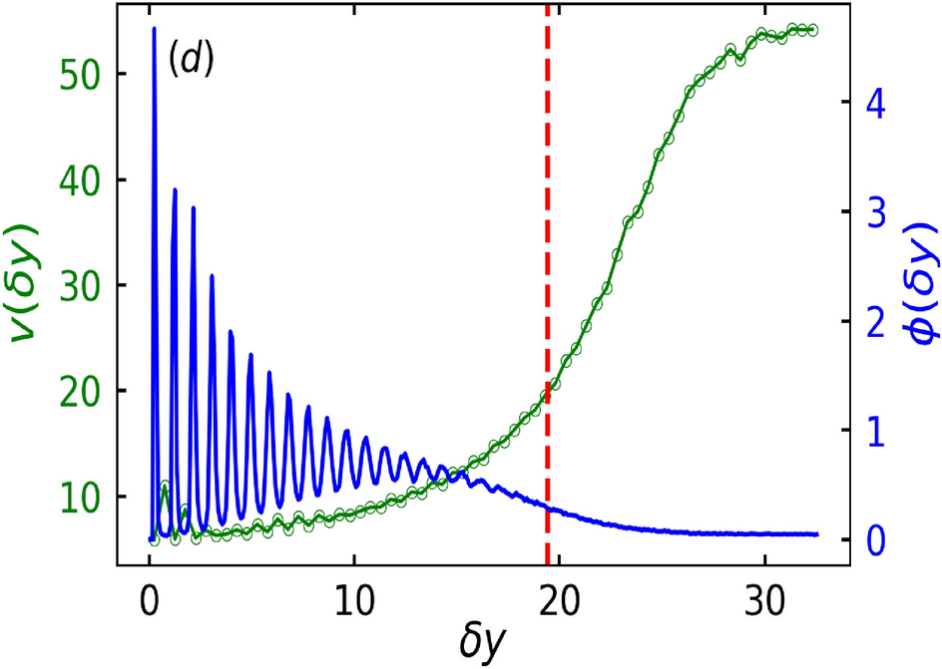
Local speed $\text {v}$ (green curve) and local area fraction $\phi$ (blue curve) as a function of distance $\delta y$ from the wall. Peaks in $\phi (\delta y)$ are indicative of layered structures. Other parameters are same as in Fig. 7.

Until now, the results pertained to the soft particle limit, whereas the hard particle limit can introduce structural changes in the aggregate as shown in Figs. 7b and 8. Figure 7c shows a snapshot of the configuration of particles in full box geometry, where the particles are colored white and cyan to belong to dense and dilute phase respectively. Zoomed configuration of the particles in magenta box (Fig. 7c) is shown in Fig. 7b. It is observed that the particles are arranged in a layered fashion. As previously stated, we can quantify the layering effect by plotting the variation of local packing fraction with distance form the wall as shown by the blue curve in Fig. 8. The $\phi$ curve shows several peaks, which are indicative of the layered organization of particles. Additionally, Fig. 8 exhibits more peaks than Fig. 5b, indicating that softness disrupts the stratification or the layering of the structure. This result is consistent with the fact that the inter-particle distance increases with increase in $\kappa$ or decrease in softness. Moreover, the local speed variation of the particles increases gradually as also observed for other layered structures. Figure 7a shows the active pressure difference curve where the large dip (red dashed line) is indicative of the average height of the cluster and smaller dip (yellow curve) is the minimum height of the clusters. Positive and negative pressure difference of the layer close to the wall shows uniform and non-uniform accumulation, as shown in the full configuration plot in Fig. 7c. This further corroborates the indicative features of the pressure difference curve previously observed for lower $\kappa$ (soft particles).

## Conclusions

In summary, this study investigates the combined effect of particle alignment and semi-confined geometry in two dimensions on the aggregate formation at the walls. We characterize the rich morphologies of particle accumulation into homogeneous state, uniform accumulation state, and non-uniform accumulation state depending upon the aggregate’s length along the wall. The system reenters into the homogeneous state upon increase in alignment strength $\Lambda$ for a given dimensionless confinement width ${\bar{L}_y}$. Interestingly, this reentrant behavior is independent of the confinement width ${\bar{L}_y}$. With decrease in ${\bar{L}_y}$, the uniform wall aggregation is found to become non-uniform for the intermediate values of $\Lambda$. The height of the aggregates display a non-monotonic dependence on $\Lambda$ for a given ${\bar{L}_y}$. Different morphologies of the system are explained using auto-correlation of the self-propulsion direction of the particles that captures the effect of increased effective collisions between the particles with increasing $\Lambda$ and decreasing ${\bar{L}_y}$. Our structural analysis reveals layer formation at low $\Lambda$ and vanishing of layers at high $\Lambda$, visualized using local packing fraction variation with distance form the walls. Moreover, the layer formation in the aggregates is identified using the active pressure difference between the normal and the tangential components. The pressure curves give insights about the average and the minimum height of the aggregates from the consecutive dips in the curves. In essence, this study advances our understanding of non-reciprocally aligned active particle dynamics in a semi-confined geometry in two dimensions.

To test our simulation results of wall aggregation and bridging in experiments, it would be interesting to study certain types of bacteria that exhibit similar reorientation mechanism that we have considered^37^. From the point of view of synthetic micorswimmers, it would be interesting to design active particles with similar alignment mechanism that is considered in this work, as in the work in Ref.^32^, in a semi-confined setup. Such experiments would be the ideal test bed to test our results. Moreover, it would be interesting to investigate the detailed aggregation kinetics of the active particles in future.

With a decrease in the steepness parameter *q*, the wall’s potential gradient becomes smoother, which can affect layers beyond the layer closest to the wall. Studying the dynamics of the system by adjusting the softness of the wall boundary could be a promising future direction for the present work. Moreover, the geometric configuration of the boundary may be configured in a ratchet-like manner to facilitate the investigation of dynamics within a corrugated channel.

## Supplementary Information

Supplementary Information.

## Figures and Tables

**Figure 9:**
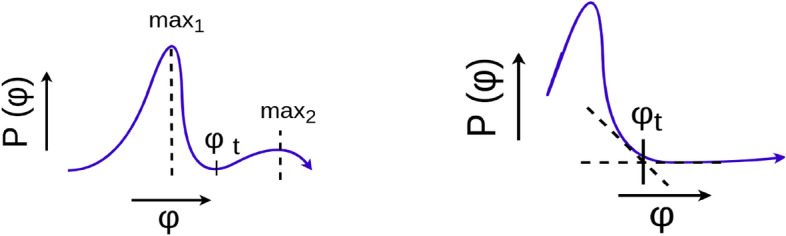
Criteria for defining target local fraction, $\phi _t$ for (left) double peaked and (right) single peak local packing fraction distribution.

## Data Availability

The datasets used and analysed during the current study available from the corresponding author on reasonable request.

## Appendix

### Quantification of dense-dilute phase

In order to identify the disks that belong to the dense or dilute region, target local packing fraction, $\phi _{t}$ is computed as follows. The simulation box, $L_x \times L_y$ is subdivided into cells of size $5 \times 5$, and the local area fraction of each cell is computed. Using this information and binsize of 1, the distribution of local area fraction is found. To compute $\phi _t$, the following approach is used, depending on whether the distribution curve is single or double peaked. For double-peaked distribution curve, $\phi _t$ corresponds to the point immediately following the first peak’s fall and preceding the second peak’s rise while for single peak distribution curve, $\phi _t$ is the point close to fall of the peak and the minima value as shown in Fig. 9. The disks belonging to the cell with local packing fraction greater than target packing fraction are tagged as dense particles otherwise they are tagged as dilute particles.

### Cluster fraction

If the value of the local area fraction of the cell is greater than the target local fraction $\phi _t$ the disks in that cell belong to the dense phase. Otherwise, they belong to the dilute phase. The fraction of particles in the cluster $N_d / N$ is now calculated by dividing the total number of disks $N_d$ in the dense phase to the total number of disks *N*. We calculate this quantity as a time-averaged quantity over steady states.

### Aggregation length and height

After tagging the particles as dense and dilute particles corresponding to the dense and dilute regions respectively, we calculate aggregation length $\mathcal {L}$ and aggregation height *h* by dividing the simulation box into cells of size $2 \times 5$. Starting from the top left cell and traversing along cells closest to the wall in x direction, we count the number of cells which are devoid of dense particles. The same process is repeated for the lower wall by starting from the bottom left cell. Computing the fraction of empty cells to the total cells along x and subtracting that from 1 gives the aggregation length.

To compute aggregation height *h* we find the maximum local height of the aggregate or cluster for each cell closest to the wall along *x*-direction for both upper and lower walls. The average of local height of these structures give the aggregation height *h*.

Aggregation length and height are further averaged over 200 steady state configurations.
